# Untargeted lipidomic analysis to broadly characterize the effects of pathogenic and non-pathogenic staphylococci on mammalian lipids

**DOI:** 10.1371/journal.pone.0206606

**Published:** 2018-10-31

**Authors:** Naren Gajenthra Kumar, Daniel Contaifer, Paul RS Baker, Kim Ekroos, Kimberly K. Jefferson, Dayanjan S. Wijesinghe

**Affiliations:** 1 Department of Microbiology and Immunology, School of Medicine, Virginia Commonwealth University, Richmond, Virginia, United States of America; 2 Department of Pharmacotherapy and Outcomes Sciences, School of Pharmacy, Virginia Commonwealth University, Richmond, Virginia, United States of America; 3 SCIEX, Concord, ON, Canada; 4 Lipidomics Consulting Ltd., Esbo, Finland; University of Liverpool, UNITED KINGDOM

## Abstract

Modification of the host lipidome via secreted enzymes is an integral, but often overlooked aspect of bacterial pathogenesis. In the current era of prevalent antibiotic resistance, knowledge regarding critical host pathogen lipid interactions has the potential for use in developing novel antibacterial agents. While most studies to date on this matter have focused on specific lipids, or select lipid classes, this provides an incomplete picture. Modern methods of untargeted lipidomics have the capacity to overcome these gaps in knowledge and provide a comprehensive understanding of the role of lipid metabolism in the pathogenesis of infections. In an attempt to determine the role of lipid modifying enzymes produced by staphylococci, we exposed bovine heart lipids, a standardized model for the mammalian lipidome, to spent medium from staphylococcal cultures, and analyzed lipid molecular changes by MS/MS^ALL^ shotgun lipidomics. We elucidate distinct effects of different staphylococcal isolates, including 4 clinical isolates of the pathogenic species *Staphylococcus aureus*, a clinical isolate of the normally commensal species *S*. *epidermidis*, and the non-pathogenic species *S*. *carnosus*. Two highly virulent strains of *S*. *aureus* had a more profound effect on mammalian lipids and modified more lipid classes than the other staphylococcal strains. Our studies demonstrate the utility of the applied untargeted lipidomics methodology to profile lipid changes induced by different bacterial secretomes. Finally, we demonstrate the promise of this lipidomics approach in assessing the specificity of bacterial enzymes for mammalian lipid classes. Our data suggests that there may be a correlation between the bacterial expression of lipid-modifying enzymes and virulence, and could facilitate the guided discovery of lipid pathways required for bacterial infections caused by *S*. *aureus* and thereby provide insights into the generation of novel antibacterial agents.

## Introduction

Infections caused by *S*. *aureus* range from skin and soft tissue infections[1], bloodstream infections[2] and infective endocarditis[3]. The genetic heterogeneity, diversity of virulence factors, and increasing resistance of *S*. *aureus* to antibiotics has garnered the interest of researchers for many decades. With greater than 5% genomic variability among strains and acquisition of virulence factors on pathogenicity islands due to phage integration [4], there are significant differences in the secretome among strains. In addition to differences in the presence or absence of virulence genes, differences in the activity of several key regulators including the accessory gene regulator (Agr) [5,6] and the SaeRS two component system[7,8] can affect the *S*. *aureus* secretome. The Agr system, which regulates virulence factor production by quorum sensing, regulates the production of extracellular toxins like alpha-, beta-, delta- hemolysin, lipase and proteases. Mutants defective in the Agr system show reduced toxin secretion and virulence in murine models of septic arthritis[6]. Studies aimed at determining the extracellular protein composition in *agr* mutants of *S*. *aureus* show that *agr* mutants had an increased retention of extracellular proteins in the cytosol [9] and reduced extracellular protease activity.

In order to colonize or invade a human host, bacteria must be able adapt to the host lipid environment and adapt to toxic lipids or modify lipids to utilize them structurally or metabolically. While many of the virulence factors produced by *S*. *aureus* are well characterized for their role in evading the immune response, less is known about virulence factors with lipid modulatory roles. Recent studies have shown that *S*. *aureus* is able to incorporate host fatty acids into its cell membrane. The resultant change in membrane fluidity and intracellular signaling [10] influences the virulence of the pathogen. Other studies reveal that staphylococci can detoxify certain fatty acids (FFA 18:0, FFA 18:1) [11–15] abundant in the mammalian microenvironment. The microenvironment of the sites colonized by *S*. *aureus* are abundant in neutral lipids like diacyl- and triacyl- glycerol and polar lipids such as derivatives of phosphatidic acid differing in the composition of the head group like phosphatidylcholine (phosphatidic acid with the choline headgroup) that make up the components of the cell membrane and the extracellular milieu. The over representation of lipases in clinical isolates[16] along with the inability of staphylococci to synthesize long chain fatty acids [17] or metabolize short chain fatty acids by beta oxidation [18] suggests that *S*. *aureus* uses novel mechanisms to modify these environments during the course of an infection and alter host and bacterial lipid homeostasis. The staphylococcal two component system, SaeRS is regulated by extracellular fatty acids and the *vfrAB* operon[19] encoding the fatty acid kinases required for the activation of free fatty acids and detoxification of growth inhibitory free fatty acids[20]. Recent work has shown that the amount of fatty acid accumulated within the cell plays a critical role in regulating the SaeRS two component system and associated virulence factors like hemolysin and coagulase[14,19]. While the sensor histidine kinase response regulator (SaeR) has no known targets for regulating the expression of lipases, there appears to be a yet unknown indirect association between the status of activation of the two-component system and the lipase activity in the extracellular milieu.

Lipases, a broad term for lipid hydrolyzing enzymes, have exquisite conformational and regioselectivity[21–23] for the substrates they modify and sometimes is an over simplification to describe this diverse group of enzymes. Extracellular lipid hydrolyzing enzymes (lipases) are processed by proteases in the extracellular milieu after secretion. This mechanism is well characterized for glycerol ester hydrolase (Geh)[24]; the major extracellular lipase expressed by *S*. *aureus* in the stationary phase of growth[9]. There is little known about the expression of other lipases by pathogenic staphylococci and their role in the virulence of the organism with the exceptions of the beta-hemolysin (Hlb) [25]; a sphingomyelinase C, and phosphatidylinositol (PI) specific phospholipase C (PI-PLC)[26]. The bulk of staphylococcal lipase research has focused on the characterization of the glycerol ester hydrolase (Geh) family, members of which hydrolyze triacylglycerols (TAG)[24,27–29]. Lipase activity in spent media from staphylococcal cultures was first reported in 1964[30]. Since then, protein purification and extraction methods have led to the identification of the well-studied lipase glycerol ester hydrolase (GehA)[31] and more recently GehB[32] and GehD[33].

Investigation of the mechanism of staphylococcal lipid modifying enzymes has been complicated by the high diversity of host lipid types and by limitations of methods used to detect and quantify host lipids. While lipases from other bacteria like *H*. *pylori* are essential to its pathogenesis[34], there is an incomplete understanding of the mechanism by which these lipases (triacylglycerol lipase and phospholipase) from *S*. *aureus* interact with host lipids and influence host cell signaling and contribute to its pathogenesis. A phospholipase C [26] reported in *Staphylococcus aureus* has specificity for the enzymatic degradation of phosphatidylinositol (PI) and the production of phosphatidic acid (PA), a mechanism central to the production of inflammation in the host cell [35,36]. Another class of lipid modifying enzymes produced by *Staphylococcus aureus*, the fatty acid kinases (FakA and FakB), have more recently been shown to play a role in the esterification of host derived fatty acids into lipids by the action of fatty acid kinases[11]. This esterification of fatty acids is important for the regulation of virulence factors [10,37] produced by this pathogen and the regulation of its biofilm phenotype[38]. Though the regulation of virulence factors due to the ability to esterify host derived fatty acids into phospholipids has been shown to be important for fitness of the pathogen in vivo[39–41], the source and the exact recipients of these host derived fatty acids remains unknown.

Considerably less is known about lipase activity in other staphylococcal species and its importance for strain specific differences in colonization of *S*. *aureus*. It will be important to determine if lipase activities are conserved among strains from different staphylococcal pulse field gel electrophoresis types and correlate it with the nature and site of infection to expand on our understanding of the role of staphylococcal lipases. This could ultimately facilitate the development of novel approaches to manage staphylococcal infections by targeting lipid metabolism [42].

Traditional methods of determining lipase activity involve the use of a differential solid medium containing tributyrin[43] (triacylglycerol analogue), egg yolk (phosphocholine analogue) [44] or triolein (triacylglycerol analogue) as substrates to indicate enzymatic hydrolysis by the formation of a zone of clearance. Other indicator-based methods that quantify fatty acid release with a change in pH and color change as an end point or use synthetically synthesized substrates to determine lipase activity do not accurately represent in vivo conditions at least in terms of the complexity of the lipid mixture. In order to determine the role of lipid modifying enzymes in the pathogenicity of microbes, it is important to determine the relevant substrates in vivo followed by their enzyme kinetics and mechanism of action. Availability of complex lipid formulations from biological matrices like heart lipid extracts and high resolution mass spectrometry-based methods to quantify these formulations has enabled the characterization of lipid composition of biological matrix[45–47], and also perform kinetic studies using mass spectrometry with short run times[48]. Additionally, advancements such as MS/MS^ALL^ based approaches in mass spectrometry (MS) on quadrupole time-of-flight (Q-TOF) instruments have improved the coverage of lipids in a single mass spectrometric run and quantification at the molecular species level [49]. Identification of the fragments that make up the molecular lipid species can then be used to reconstruct the true molecular lipid information required to infer biologically relevant modulations and gain insight into the mechanisms by which pathogens interact with host lipids.

In this study, we employed spent media from cultures of four strains of *S*. *aureus*, a clinical isolate of the normally commensal *S*. *epidermidis*, and a strain of the non-pathogenic *S*. *carnosus* [50,51] and compared lipid modifying activity in the samples by using bovine heart extract as a model substrate. Through the application of a shotgun lipidomics approach[49], we build on classic plate based assays [52] and kit based methods[53] used to investigate the lipase activity of these bacteria in greater detail. We demonstrate the ability of shotgun lipidomics[49] to corroborate previously reported lipase activities, and detect these activities in spent media from staphylococcal cultures. In addition, the results suggest that compounds with previously unreported lipase activities are secreted by commensal and pathogenic staphylococci.

## Materials and methods

### Experimental design and workflow

Heart Total Extract (**171201C)** was purchased from Avanti Polar Lipids, Inc. Strains used in this study were obtained from the following sources (Table 1);*S*. *aureus* JE2(USA300) [26] (BEI Resources, Manassas, VA), *S*. *carnosus* TM300[51,54] (Friedrich Gotz University of Tübingen, Tübingen, Germany).

**Table 1:** Description of strains used in the study.

Strain	Description	Ref
***S*. *aureus*** **JE2**	USA300, community-acquired, methicillin resistant (MRSA) clinical wound isolate	[26]
***S*. *aureus*** **MN8**	USA200, community-acquired, methicillin sensitive (MSSA) toxic shock syndrome isolate	[55]
***S*. *aureus*** **COL**	*agr-*, hospital-acquired MRSA, historical wound isolate	[56]
***S*. *aureus*** **Newman**	Constitutive *saeS*, MSSA, secondary infection of *Mycobacterium tuberculosis* osteomyelitis	[4,7,57,58]
***S*. *epidermidis*** **RP62A**	Clinical isolate from catheter-related infection	[54]
***S*. *carnosus*** **TM300**	Non-pathogenic isolate deemed safe for use in food industry	[51,54]

### Preparation of spent media from bacterial cultures

All strains of staphylococci were cultured for 6 hours in 3ml Tryptic Soy Broth (TSB) in snap cap tubes. O.D._600nm_ determined and adjusted to the lowest O.D. (*Staphylococcus carnosus*) with TSB. Bacteria were pelleted by centrifuging at max speed for 1 minute in a microcentrifuge at room temperature. The cell free supernatant was filtered through a 0.45μm filter. All bacterial spent media samples were diluted 1:1 with sterile phosphate buffered saline (PBS) prior to performing lipase assays. Heat inactivated controls of the spent media samples were prepared by heating the supernatant to 80 ^o^C in a heat block for 20 minutes prior to performing 1:1 dilution used in the assays.

### Egg yolk agar lipase activity

Egg yolk agar plates were prepared as manufacturer instructions in TSB. Egg yolk agar plates were made by autoclaving 15g of TSB (BD Bacto Tryptic Soy Broth) and 7.5g Bacto agar (BD Bacto Dehydrated Agar) combined with 500ml of deionized water. Once cooled to approximately 50°C, 50 ml of egg yolk agar emulsion (HI media Egg Yolk emulsion HiMedia Laboratories) was added before the agar was poured into petri dishes. A 10 μL loop was used to streak bacterial cultures (described above) onto the agar. Plates were incubated for 20 hr at 37 ^0^C and the zone of clearance was measured using ImageJ.

### Lipase activity in spent media

Filter sterilized spent media from exponential cultures grown in TSB for 6 hr were used to determine lipase activity analyzed using the QuantiChrom Lipase Assay Kit (BioAssay Systems). Absorbance measure at 412nm at 10 min and 20 minutes was used to determine the lipase activity of the crude spent media in U/L as per manufacturer instructions[53].

### Preparation of heart lipid extract for assays

0.6mg (500μl of 1.25mg/ml stock) of total heart extract was dried under vacuum in a speedvac for 30 minutes. Dried lipids were suspended in 3 ml of sterile PBS. The mixture was sonicated at the lowest power using a probe sonicator (Fisher Scientific Series 60 Sonic Dismembrator Model F60 model # 22141) until the lipids were completely suspended and a homogenous milky white suspension was obtained. 200 μl of this suspension was dispensed into auto sampler vials. To this 50 μl of the 1:1 diluted bacterial supernatant or PBS was added and incubated at 37°C overnight.

### Lipid extraction

Lipids were extracted using a modified Bligh and Dyer method [59]. Lipids from the 250 μl reaction volume were extracted using 1 ml methanol and 0.5 ml of chloroform followed by addition of 10 μl of SPLASH lipidomics internal standard mix (Avanti Polar Lipids) and incubated at room temperature for 10 minutes. 1 ml of LCMS grade water and 1 ml of chloroform was added to extraction mixture. This mixture was vortexed for 10 seconds and centrifuge in a pre-cooled centrifuge for 20 minutes at 3500 rpm. From the phase-separated mixture thus obtained, the bottom hydrophobic organic phase (bottom phase) was transferred into a fresh glass tube using a pasteur pipette. The organic phase containing the lipids was dried under a stream of nitrogen and lipids were resolubilized in 250ul of the infusion solvent (MeOH/CH_2_Cl_2_, 50:50 containing 5mM ammonium acetate).

### MS/MS^ALL^ mass spectrometric analysis

MS/MS^ALL^ mass spectrometric analysis [60] was performed on a Sciex Triple-TOF 5600+ via direct infusion using a Shimadzu SIL-20ACXR at a flow rate of 9ul/min over 25 minutes. The samples were infused using a flow gradient. Initially, the infusion flow rate was 9 μl/min. After 8 min, the flow rate was increased to 100 μl/min for 2 min to rinse the flow path. After which, the flow was returned to 9 μl/min and the system pressure re-equilibrated. To minimize carryover, PEEKSil tubing was used to connect the autosampler directly to the mass spectrometer. Normal PEEK tubing has significant carryover issues with infusion Lipidomics. The experimental parameters used to analyze lipids in the mass range (*m/z* 100–1200) were, positive ionization mode: CUR 30, GS1 16, GS2 40, TEM 150, accumulation time 5000 ms, CAD 4, CE 38, CES 5, DP 80 and negative ionization mode: CUR 30, GS1 16, GS2 40, TEM 100, accumulation time 3000 ms, CAD 4, CE -45, CES 5, DP -80.

#### Identification of molecular lipid species and quantification

Lipid species based on precursor fragment ion pairs were determined using a comprehensive target list in LipidView (Sciex). Lipid species identification was performed using; the mass tolerance of 0.3 in MS and 0.5 in MS/MS, s/n of 5 and % peak intensity > 2 for positive ion mode, and 0.3 in MS and 0.5 in MS/MS, s/n of 5 and % peak intensity >2 for negative ion mode. Identified lipid species list was filtered to include all lipid species with greater than 30 counts per second and less than 75% missing values in all samples. Quality control on this list was performed by ranking the detected in both modes and ensuring that the top 10 lipid species in both modes are the same. Molecular lipid species were determined from this filtered list and the total abundance of the molecular lipid was determined by summing the peak intensity of the contributing fragments[46]. Lipid classes included for statistics and downstream analysis were cholesterol ester (CE), sphingomyelin (SM), diacylglycerol (DAG), triacylglycerol (TAG) and ceramide (Cer) from positive mode and phosphatidylcholine (PC), phosphatidylethanolamine (PE), phosphatidylglycerol (PG), phosphatidylinositol (PI), phosphatidylserine (PS) and lysophosphatidylcholine (LPC), lysophosphatidylethanolamine (LPE), lysophosphatidylglycerol (LPG), lysophosphatidylinositol (LPI), lysophosphatidylserine (LPS) in the negative mode. The molecular lipid identifications in both modes were combined to make one comprehensive list and the Mol% of each lipid was determined by dividing the peak intensity by the sum of all lipids in the sample and multiplying by 100 [46]. Non-parametric statistics was performed to determine significant lipids followed by Benjamini Hochberg correction (BH). Lipids were determined to be significant only if the adjusted p-value after BH correction was less 0.05. The final naming of the lipids was performed as per the nomenclature of the lipid class followed by the fatty acid species in increasing order of carbon chain and unsaturation [61,62].

### Statistical analysis

All statistical analysis were performed using R 3.3.0 [63]. Wilcoxon Rank-Sum test was used for all group analysis and Kruskal-Wallis followed by Benjamini Hochberg correction was performed for multiple comparisons with significance at p<0.05. Plots were prepared using packages pheatmap[64] and ggpubr[65].

## Results

### Experimental workflow

**Fig 1:**
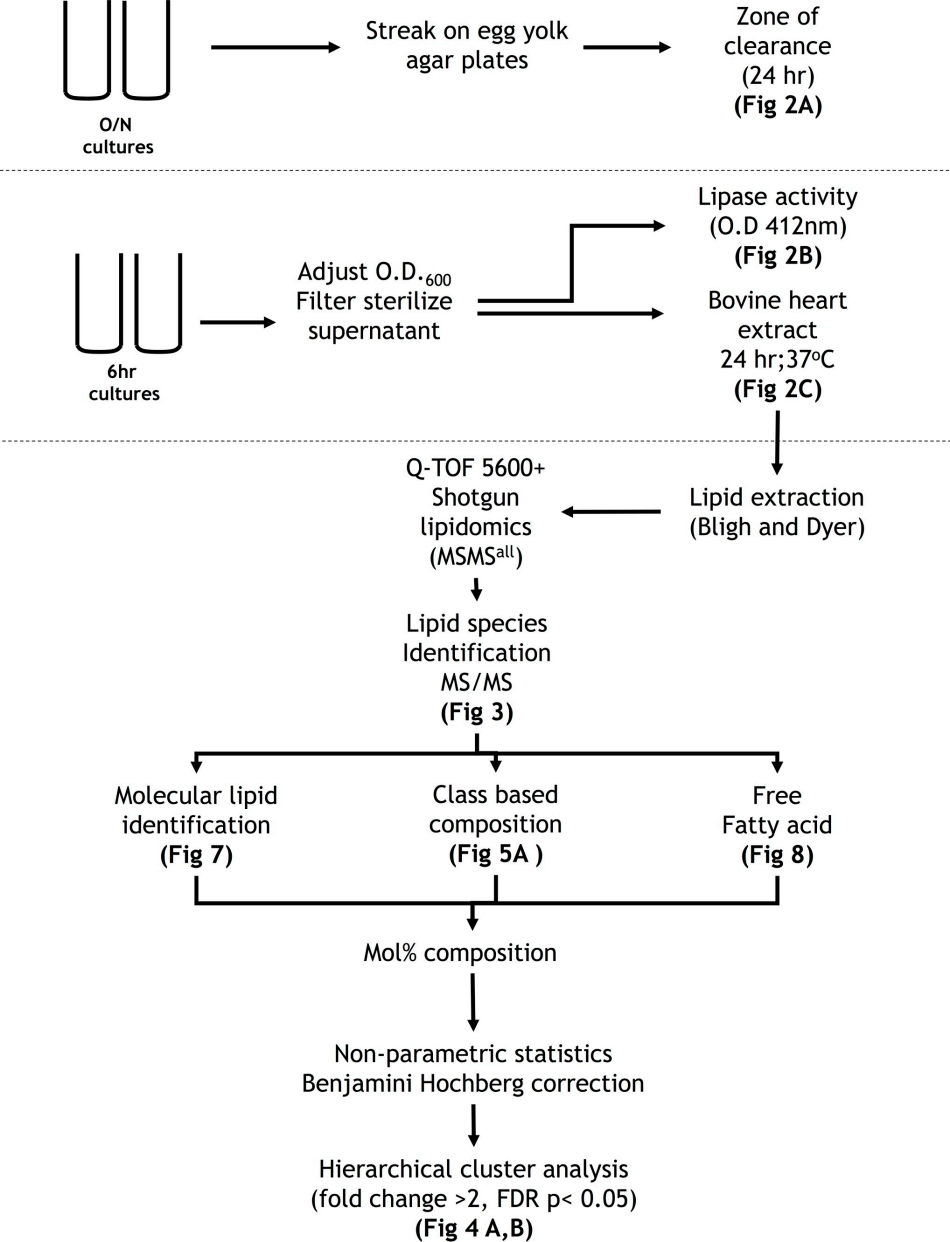
Experimental and analytical workflow to investigate lipase activity in spent media from cultures of staphylococcal strains.

Three assays used to investigate lipase activity in the spent media from staphylococcal cultures are illustrated in (Fig 1). It summarizes the analytical workflow for the shotgun lipidomics approach and statistical approach to arrive at the results.

### Traditional lipase assays detect lipase activity in spent media from *S*. *aureus JE2 and MN8*

**Fig 2:**
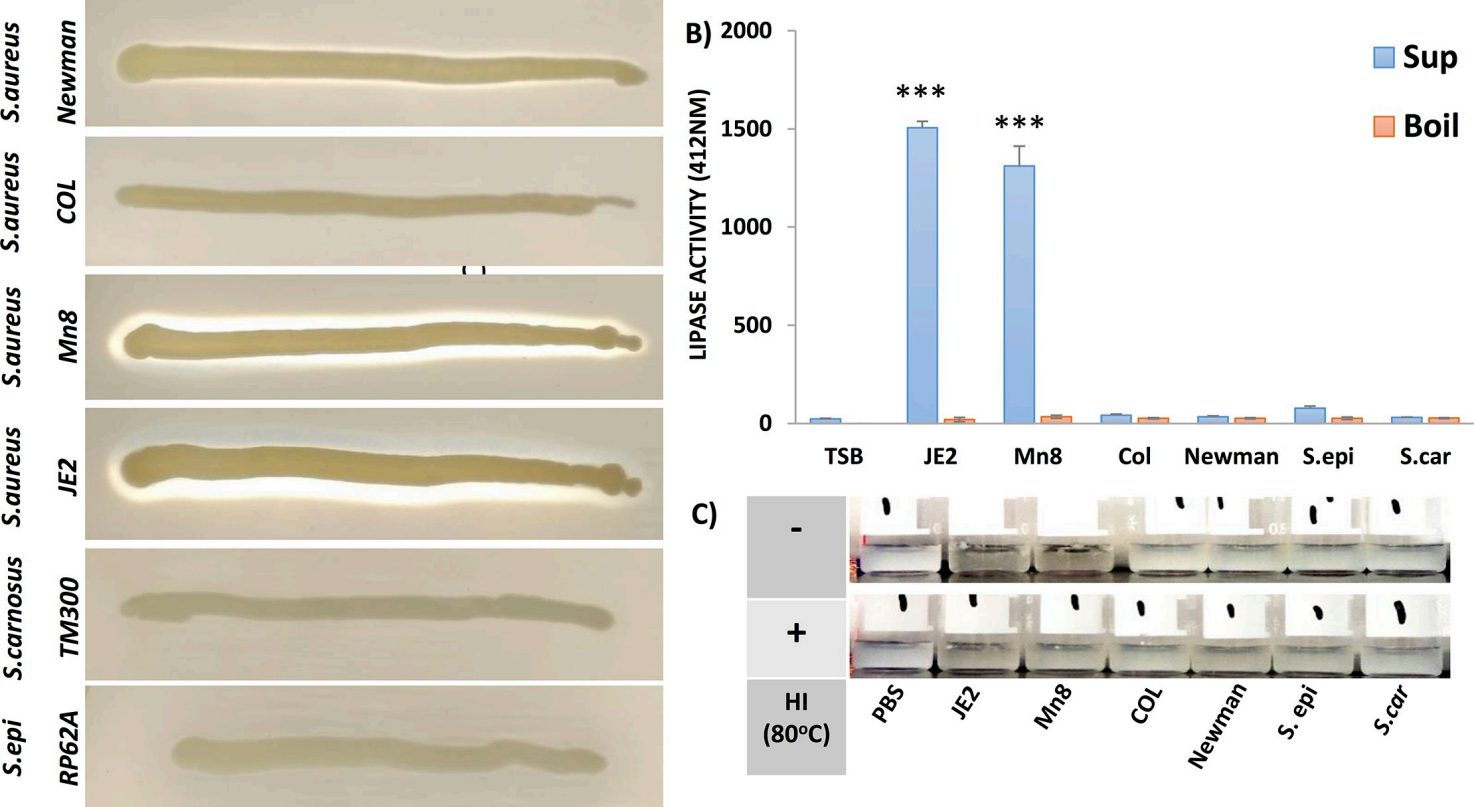
Lipase activity in spent media. A) Formation of zone of clearance on 10% egg yolk Tryptic Soy Agar (TSA) plates around streaks of staphylococcal strains incubated overnight at 37°C. B) Lipase activity in filter sterilized spent media samples diluted (1:1) with PBS from 6 hour cultures was quantified using Quantichrom Lipase assay kit (A_412nm_). C) Clearance of the 0.6 mg heart extract after overnight incubation with the spent media from staphylococcal strains with heat inactivation (+) and without heat inactivation (-). Error bars in (B) are standard deviations of an experiment done in triplicates. *** p< 0.0001 Kruskal-Wallis. TSB; TSB control, PBS; PBS control, *JE2; S*. *aureus JE2*, *MN8*: *S*.*aureus MN8*, *COL; S*. *aureus (agr-)*, *Newman; S*. *aureus Newman (constitutive saeS)*, *S*. *epi; S*. *epidermidis RP62A*, *S*. *car; S*. *carnosus TM300*.

Overnight cultures of *S*.*aureus* (JE2, MN8, COL, and Newman), *S*. *epidermidis* (EPI) and *S*. *carnosus* (CAR) were streaked on Tryptic soy Agar containing 10% egg yolk emulsion and incubated at 37°C. Overnight incubation resulted in the formation of zones of clearance around *S*. *aureus* (JE2 and MN8) (Fig 2A) but zones of clearance around *S*. *aureus* COL and Newman, *S*. *epidermidis*, or *S*. *carnosus* were not as readily observable, even after prolonged period of incubation at 37°C for up to two days (Fig 2A). The formation of the zone of clearance is due to the degradation of the lipids in the egg yolk emulsion and is indicative of lipase activity. Lipase activity in filter sterilized spent media from 6 hours cultures diluted 1:1 in 1X PBS was quantified using a DTNB based Lipase assay kit[53]. Activity recorded by measuring absorbance at 412nm indicated the presence of lipase activity in spent media from *S*. *aureus* JE2 and MN8, less activity associated with Newman, and little or no detectable lipase activity in spent media from the other strains [53] (Fig 2B). Heat inactivation (HI) of the media samples resulted in complete inactivation of lipase activity in the spent media (Fig 2B). Overnight incubation of spent media from JE2 and MN8 with the heart extract, resulted in clearance of the heart extract and recapitulated the pattern observed on egg yolk agar plates but clearance was not observed in samples containing spent media from the other strains, heat inactivated (HI) media, or PBS alone (Fig 2C).

### Confirmation of lipid identification based on tandem MS spectra

**Fig 3:**
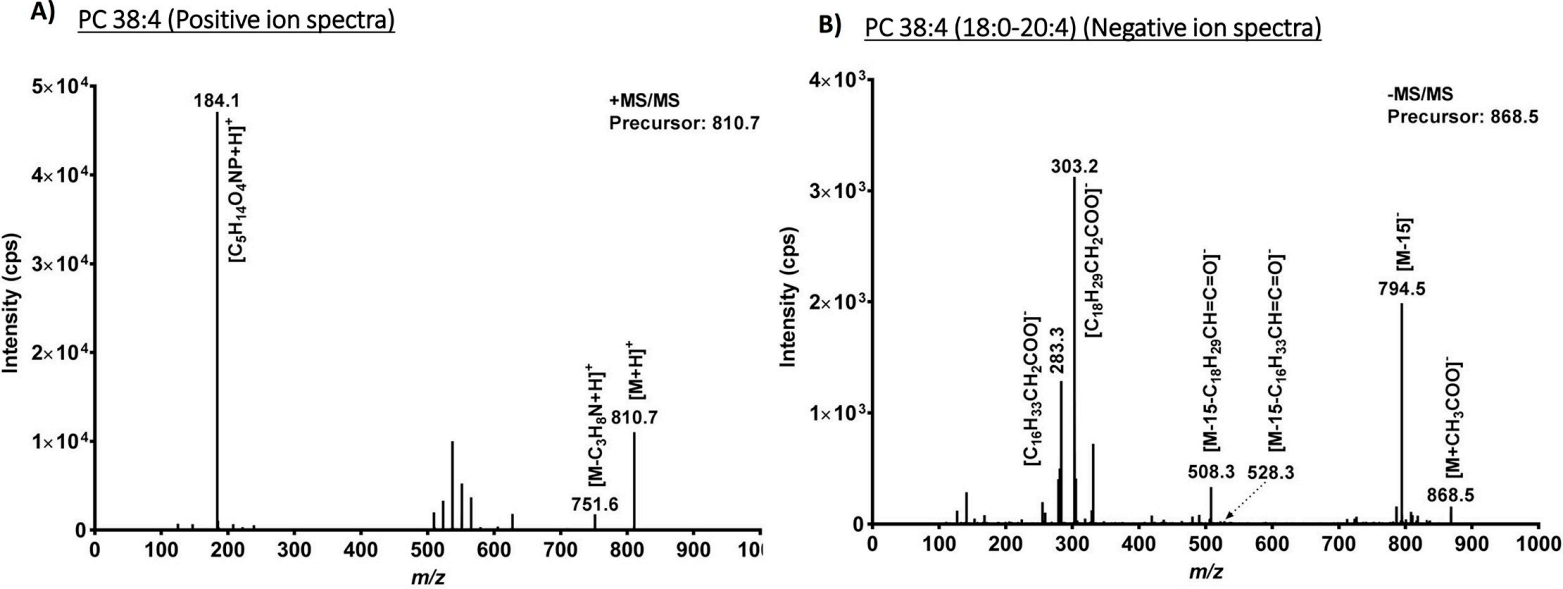
Confirmation of lipid identification. Confirmation of PC 38:4 in positive (A) and negative ion mode (B), respectively. Upon fragmentation of the precursor ion of *m/z* 810.7 in positive ion mode the phosphatidylcholine ion corresponding to PC head group, mz/184.1 is generated [67]. In negative ion mode the corresponding acetate adduct of *m/z* 868.5, generates the expected fragment ions corresponding to neutral loss as ketenes and acyl anions of *m/z* 283.3 (FA 18:0) and *m/z* 303.2 (FA 20:4) respectively. From the precursor ion masses (*m/z* 868.5) and generated fragment ions (*m/z* 283.3 and *m/z* 303.2) the lipid can be confirmed as a PC 18:0–20:4 (PC 38:4 as sum formula)[61].

To ensure correct lipid identifications for downstream lipidomics analysis, the lipids were analyzed in both positive and negative modes of ionization[66]. For example, the phosphatidylcholine (PC) 38:4 *m/z* 810.7 in concert with the generation of the phosphocholine head group *of m/z* 184.1 in MS/MS (Fig 3A) confirmed detection in the positive mode and *m/z* 868.5 in negative ion mode was identified as an acetate adduct of phosphatidylcholine PC 18:0–20:4 (PC 38:4) (molecular species; 18:0–20:4, based on fragment ion masses 283.3 (FA 18:0) and 303.2 (FA 20:4)) (Fig 3B). A similar approach was adopted for other lipids reported.

### Changes to mammalian lipids differ between species and strains

**Fig 4:**
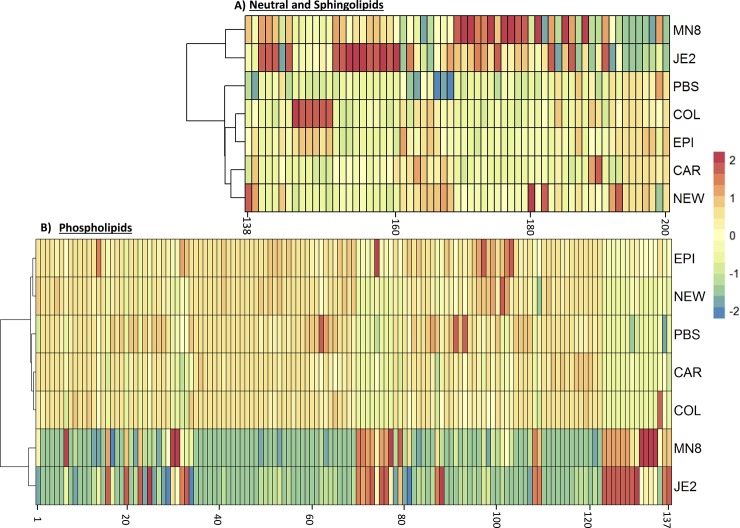
**Unidirectional Hierarchical clustering of significant lipid species (A) Neutral lipids (B) Phospholipids.** PBS; PBS control, *JE2; S*. *aureus JE2*, *MN8*: *S*. *aureus MN8*, *COL; S*. *aureus (agr-)*, *NEW; S*. *aureus Newman (constitutive saeS)*, *EPI; S*. *epidermidis RP62A*, *CAR; S*. *carnosus TM300*.

Mol% data representing the abundance of a molecular lipid in each sample was used for the analysis. We determined if the profiles of lipid modulation for *S*. *aureus* JE2 and MN8 were similar to each other and distinct from the other strains. Using a unidirectional hierarchical clustering approach to cluster each of the strains based on the mean Mol% abundance of lipid species determined to be significant (Kruskal-Wallis p<0.05 and Benjamini Hochberg p<0.05) (Fig 4A and S1 Fig, S2 File), we were able to identify the similarities between pathogenic *S*. *aureus* JE2 and MN8. The clustering approach separated *S*. *aureus* strains JE2 and Mn8 from the other strains based on their ability to modify neutral-, sphingo- and phospho-lipids alike (Fig 4A and 4B; S1 and S2 Figs).

### *S*. *aureus* JE2 and MN8 selectively modify certain lipid classes

**Fig 5:**
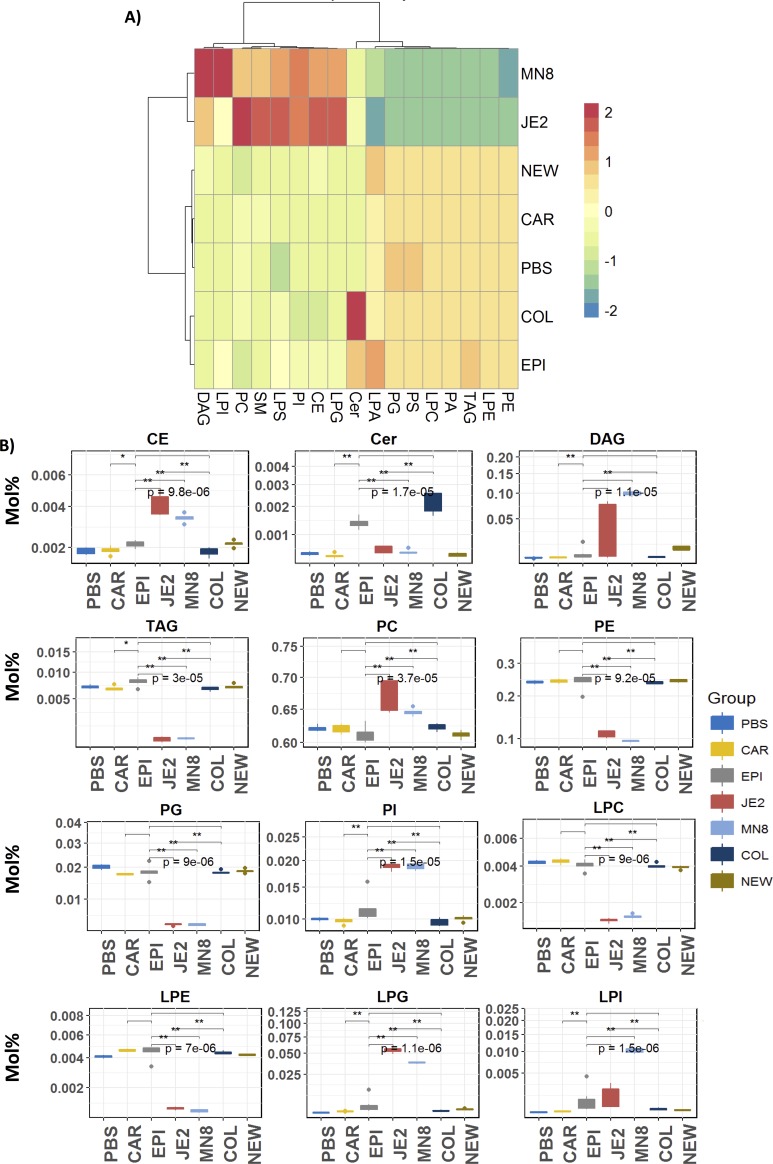
Modulation of select lipid classes by spent media from staphylococcal strains. CE; Cholesteryl ester, Cer; Ceramide, DAG; Diacylglycerol, TAG; Triacylglycerol, PC, phosphatidylcholine, PE; phosphatidylethanolamine, PG; phosphatidylglycerol, PI; phosphatidylinositol, LPE; lysophosphatidylethanolamine, LPG; lysophosphatidylglycerol, LPI; lysophosphatidylinositol, LPC; lysophosphatidylcholine. *PBS; PBS control*, *SA; S*. *aureus JE2*, *MN*: *S*. *aureus MN8*, *COL; S*. *aureus (agr-)*, *NEW; S*. *aureus Newman (constitutive saeS)*, *EPI; S*. *epidermidis RP62A*, *CAR; S*. *carnosus TM300*.

Bidirectional hierarchical clustering based approach was used to discern the effect of lipase activity from each of the strains on specific lipid classes (Fig 5A). To determine the classes of lipids specifically targeted by *S*. *aureus* lipases, we examined the effect of the treatments at the lipid class composition level. The sum lipid class composition was determined for each sample and the replicates were averaged per treatment group. Hierarchical clustering to determine the specificity of the strains for the classes of lipids present in the heart extract. This clustering approach separated *S*. *aureus* strains JE2 and MN8 independent from the other strains (Fig 5A). Lipolytic activity from *S*.*aureus* JE2 and MN8 was specific for the classes of phosphatidic acid, phosphatidylethanolamine (PE), phosphatidylglycerol (PG), phosphatidylserine (PS), triacylglycerol, lysophosphatidylcholine (LPC) and lysophosphatidic acid (LPA) while an increase in abundance of lipid classes of cholesteryl esters (CE), phosphatidylinositol, diacylglycerol (DAG), Ceramide (Cer) and lysophosphatidylglycerol (LPG) was observed. Additionally, this approach also separated the lipid classes into two distinct clusters based on lipid classes that were significantly degraded (triacylglycerol, lysophosphatidylethanolamine, phosphatidylethanolamine, lysophosphatidylcholine, phosphatidic acid, phosphatidylserine, phosphatidylglycerol, lysophosphatidic acid) and those that were increased (diacylglycerol, lysophosphatidylinositol, cholesteryl ester, phosphatidylinositol, lysophosphatidylserine, sphingomyelin, phosphatidylcholine, lysophosphatidylglycerol and ceramide) as a result of lipid metabolism (Fig 5A). There was a significant increase in ceramides in samples treated with spent media from *S*. *epidermidis* and *S*. *aureus* COL and decreases in the classes of triacylglycerol, phosphatidylethanolamine, phosphatidylglycerol, lysophosphatidylcholine and lysophosphatidylethanolamine, suggesting the presence of lipase activities specific for these classes of lipids in the spent media for *S*. *aureus* JE2 and MN8 (Fig 5B).

### Modulation of specific neutral and sphingolipids

**Fig 6:**
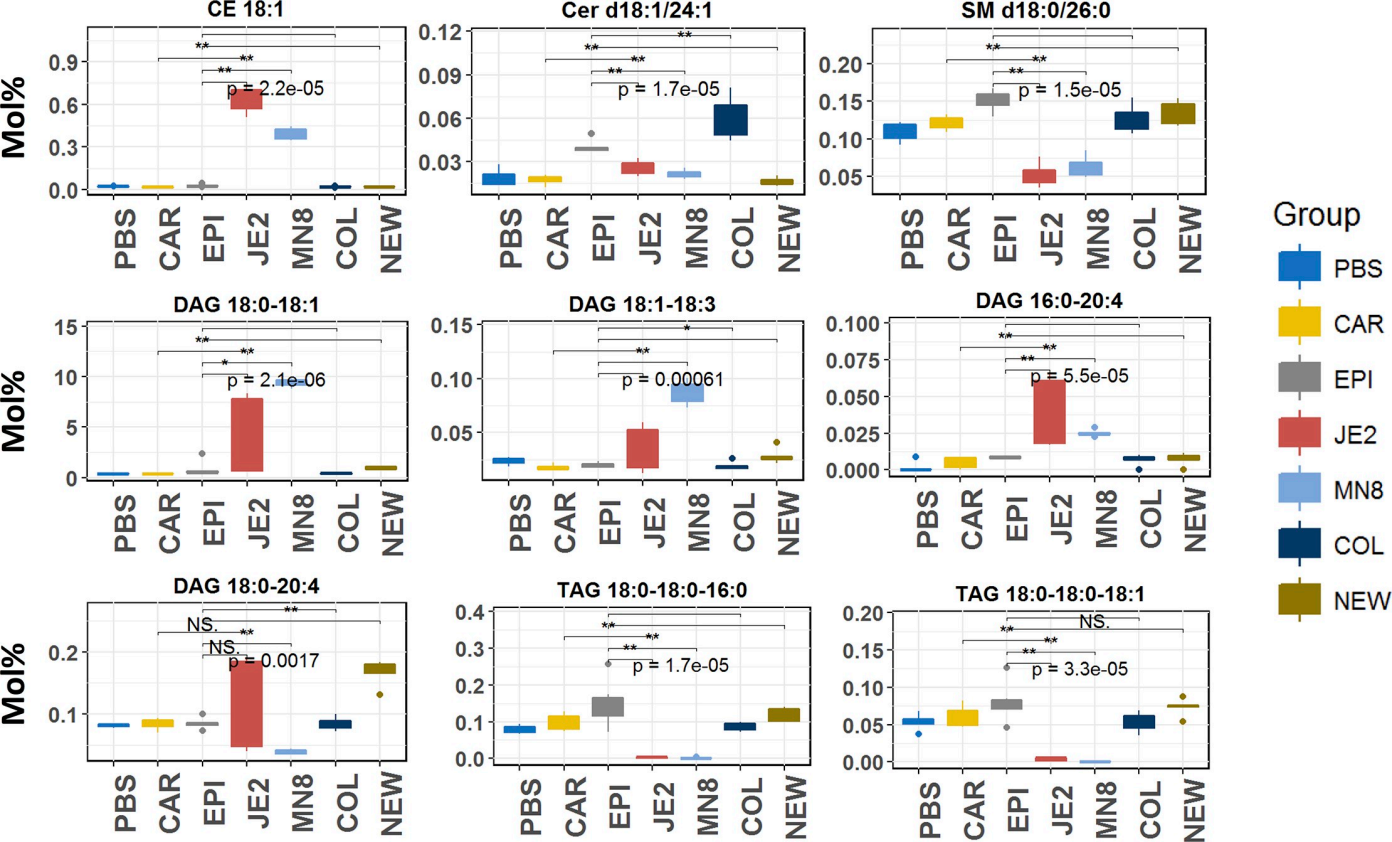
Significant neutral and sphingolipids modified by spent media from staphylococcal strains. Cholesteryl esters (CE), Ceramide (Cer), Sphingomyelin (SM), Diacylglycerol (DAG), Triacylglycerol (TAG). PBS; PBS control, SA; *S*. *aureus* JE2, MN: *S*. *aureus* MN8, COL; *S*. *aureus* (*agr*-), NEW; *S*. *aureus* Newman (constitutive *saeS*), EPI; *S*. *epidermidis* RP62A, CAR; *S*. *carnosus* TM300. Y-axis Mol% composition of lipid classes in heart extract.

To further interrogate the modulations of lipids within the lipid classes affected by the different staphylococci (Fig 6) we focused on individual lipid species that were modulated by crude spent media from each of our strains. Increases in cholesteryl esters after treatment with *S*. *aureus* JE2 and MN8 was consistent across all of the significant species and the increase in CE 18:1 is representative of the trend for other significant cholesteryl esters (Fig 5). Spent media from *S*. *aureus* JE2 and MN8 had no effect on ceramides; a major component of the skin[68,69], while treatment with spent media from *S*. *aureus* COL (COL) and *S*. *epidermidis* (EPI) resulted in a uniform increase similar to Cer d18:1/24:1 in all species of ceramides found to be significant relative to PBS controls. Sphingomyelin (SM), a major component of mammalian cell membranes and found to be associated with lipid rafts was mostly unaffected with the exception of a reduction in SM d18:0/26:0 in the *S*. *aureus* JE2 and MN8 treated groups. Triacylglycerols, for which only the lipids with all three fragments identified from the MS/MS spectra were included (i.e. TAG 18:0–18:0–18:1 (TAG 54:1)) (Fig 6), were uniformly degraded only in the *S*. *aureus* JE2 and MN8 treated groups with no changes observed by other spent media or heat inactivated controls. Diacylglycerols were differentially modulated only by *S*. *aureus* JE2 and MN8 and there was no conserved pattern for hydrolysis.

### Modulation of phospholipids

**Fig 7:**
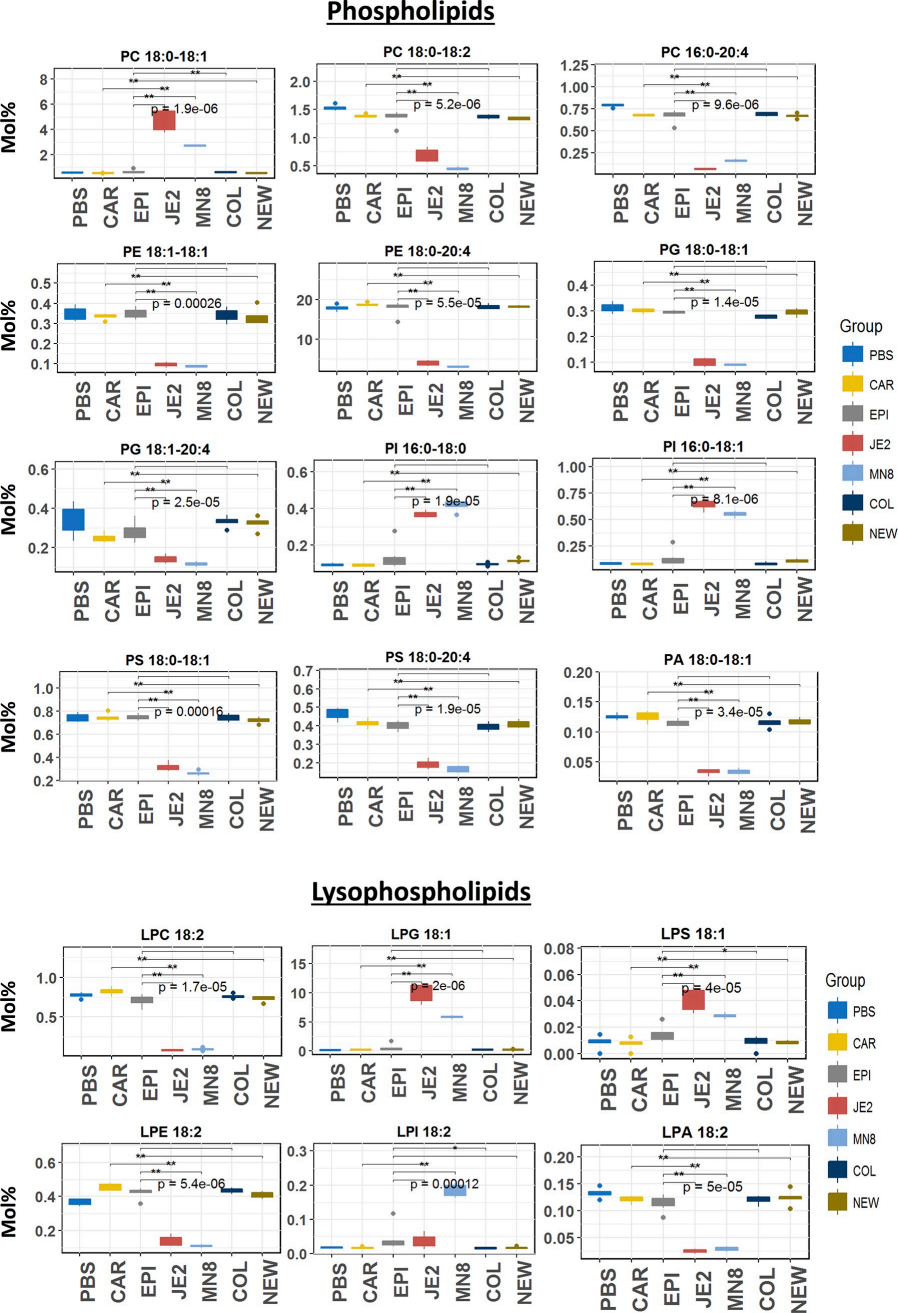
Significant phospholipids modified. Modulation of the classes of phosphatidic acid (PA), phosphatidylcholine (PC), phosphatidylethanolamine(PE), phosphatidylinositol (PI), phosphatidylserine (PS), lysophosphatidylcholine (LPC), lysophosphatidylglycerol (LPG), lysophosphatidic acid (LPA), lysophosphatidylinositol (LPI). *PBS; PBS control*, *SA; S*. *aureus JE2*, *MN*: *S*. *aureus MN8*, *COL; S*. *aureus (agr-)*, *NEW; S*. *aureus Newman (constitutive saeS)*, *EPI; S*. *epidermidis RP62A*, *CAR; S*. *carnosus TM300*. *Y-axis mol% composition of lipid classes in heart extract*.

While analyzing the modulation in phospholipids, it was observed that most increases resulting from enzymatic activity in the crude spent media was in lipids associated with oleic acid (18:1) such as PI 16:0–18:1 (PI 34:1) (Fig 7), in classes previously shown to be increased in *S*. *aureus* JE2 and MN8 treated groups (Cholesteryl esters (CE), phosphatidylcholine, phosphatidylinositol, lysophosphatidylglycerol) (Fig 5A). In all significantly modulated phospholipids (phosphatidylcholine, phosphatidylethanolamine, phosphatidylglycerol, phosphatidylinositol, phosphatidylserine) there was a uniform reduction in lipids carrying the inflammatory arachidonic acid (20:4). All lyso-lipids with the exception of the class of lysophosphatidylglycerol, lysophosphatidylinositol and lysophosphatidylserine were significantly degraded in the *S*. *aureus* JE2 and MN8 treated groups, which showed greater than 2-fold increases. The increases in these lyso lipid classes were detected in and not restricted to lyso lipids containing oleic acid (18:1) at either the sn-1 or sn-2 position.

Phospholipids; phosphatidylcholine and phosphatidylethanolamine, the most abundant lipids in the plasma membrane of the mammalian host cell and gram-negative bacteria, a reduction was observed independent of fatty acid composition in PE. Among phosphatidylcholines a uniform reduction in all lipid species with the exception of PC 18:1–20:3 (PC 38:4)and PC 18:1–20:4 (PC 38:5) (Fig 7) that showed a significant increase after treatment with spent media from *S*. *aureus* JE2 and MN8. No similar increases were observed among PE. Similarly, as expected, a reduction of all molecular species was observed among phosphatidylglycerol (PG 36:1 (18:0–18:1) (Fig 7). However, among PI an increase was associated with 16 and 18 carbon chain fatty acid containing lipids (PI 16:0–18:1 (PI 34:1))). A uniform reduction was observed among the molecular lipid species of phosphatidylserine; another major component of the mammalian cell membrane and a marker of apoptosis.

### Treatment with spent media from *S*. *aureus* releases free fatty acids

**Fig 8:**
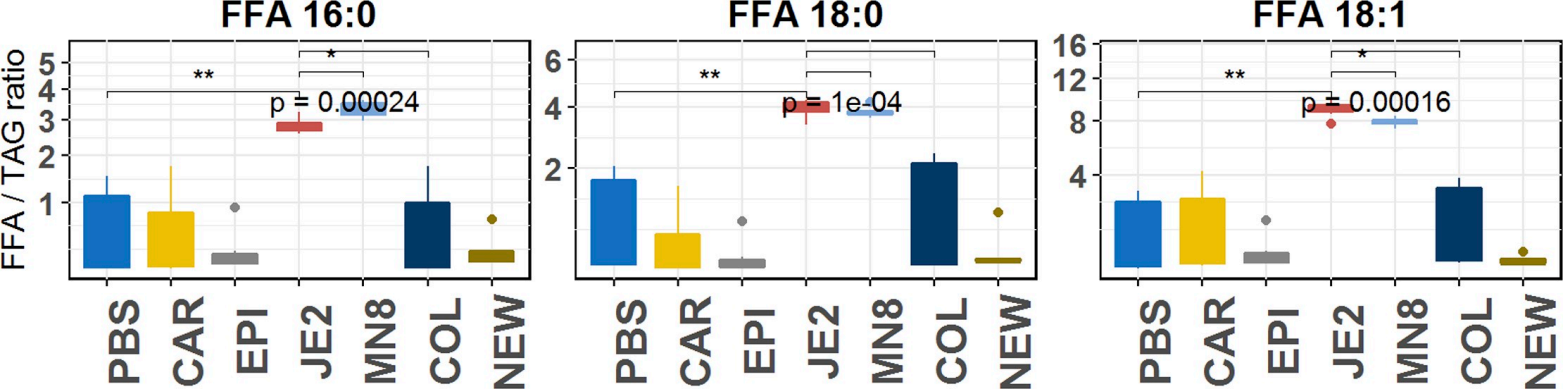
Free fatty acid release by *strains of S*. *aureus (JE2 and Mn8)*. PBS; PBS control, SA; S. aureus JE2, MN: S.aureus MN8, COL; S. aureus (agr-), NEW; S. aureus Newman (constitutive saeS), EPI; S. epidermidis RP62A, CAR; S. carnosus TM300. Y–axis relative abundance of free fatty acid normalized to total triacylglycerol content in each sample (FFA/TAG ratio).

Degradation of fatty acid containing lipids would result in the increase in the free fatty acid content in the samples. To normalize the free fatty acids detected, we determined the total free fatty acid content, normalized to triacylglycerol (FFA/TAG), in heart lipid extract treated with spent media from the staphylococci. We observe an increase in the relative abundance of medium chain fatty acids (16–18 carbon) when compared to *S*. *epidermidis* and *S*. *carnosus* treated groups (Fig 8). Significant increases were observed in FA 16:0, FA 18:0 and FA 18:1 with spent media from *S*. *aureus* JE2 and MN8 (Fig 8).

### Modeling lipase activity in staphylococcal spent media

**Fig 9:**
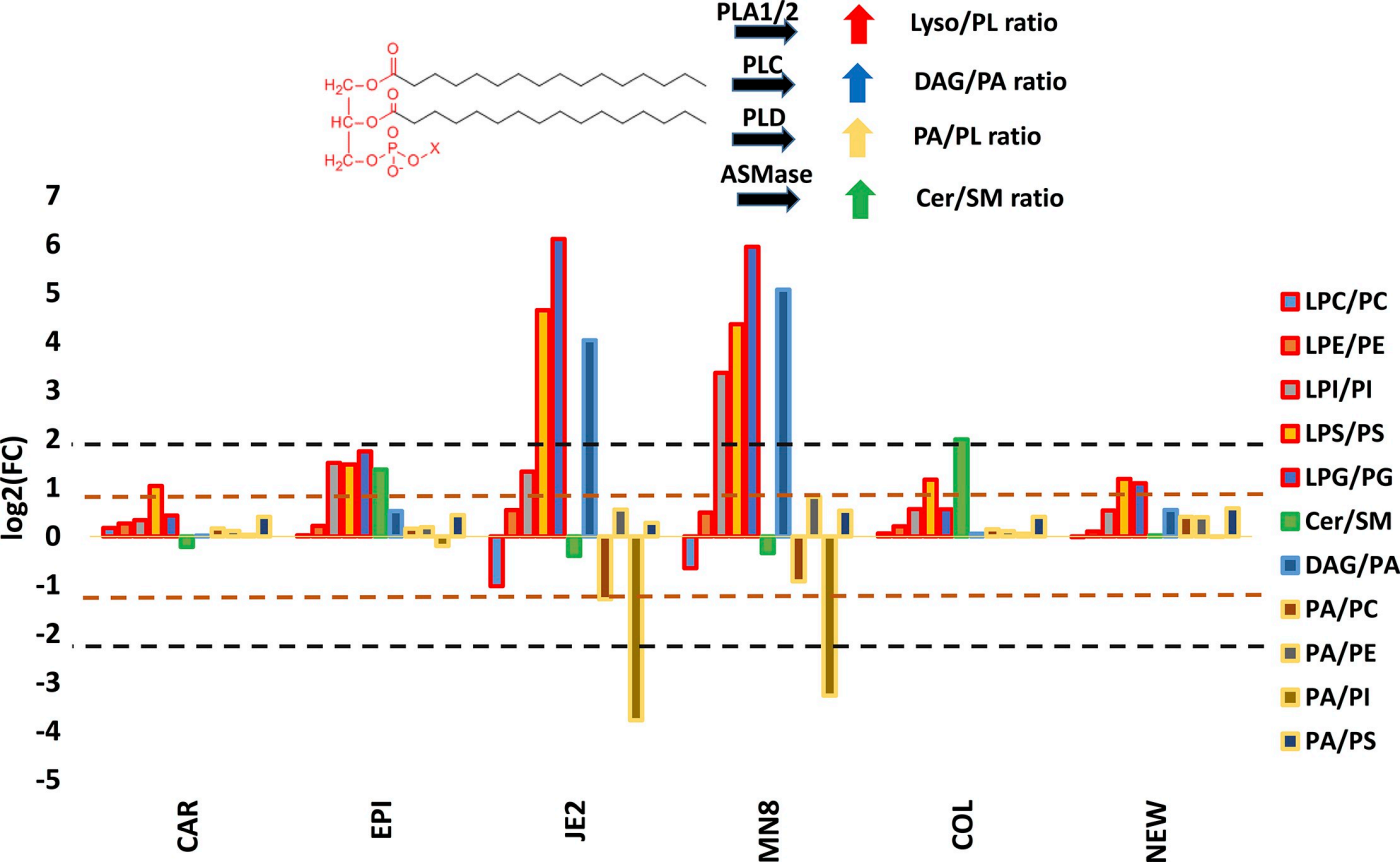
Discernable lipase activities in spent media. The bar graphs represent log2(fold change) in the respective ratios relative to PBS controls to determine if the lipids in the denominator are likely to serve as a source for the production of the lipids in the numerator. A positive value would suggest that the numerator was in a higher concentration relative to the denominator. The fold change is relative to untreated PBS controls and depicts the impact of the treatment. Border of each bar is assigned according to the color of the arrow representing the kind of lipase activity represented by the ratio. Lyso- species are LPC, LPE, LPI, LPS. Phospholipids (PL) are designated as; PA, PC, PE, PG, PI, PS. SA; *S*. *aureus* JE2, MN: *S*.*aureus* MN8, COL; *S*. *aureus* (*agr-*), NEW; *S*. *aureus* Newman (constitutive *saeS*), EPI; *S*. *epidermidis* RP62A, CAR; *S*. *carnosus* TM300.

Since the spent media from different staphylococci contain a variety of lipases with defined specificities for phospholipids, we sought to summarize their activity based on our comprehensive lipidomics approach. We expand on a method previously described to determine the presence of PLA1/2 activity in intact serum by MALDI-TOFMS[70] using the richness of the data acquired in our study. We calculated the ratio of lysophospholipids to phospholipids in a lipid class in each treatment and then determine the fold change relative to the PBS control to normalize for the natural abundance of these lipids in the matrix. In doing so, we determined whether it was likely that the phospholipids (denominator) served as a source for the production of lyso lipids (numerator) as a result of phospholipase A1/A2 activity. For example, to determine if phosphatidylcholine-specific phospholipase A1/A2 (PC- PLA1/2) activity is present, we determined the ratio of lysophosphatidylcholine to phosphatidylcholine. Since diacylglycerols can be produced from multiple sources, we focused on determining if there was any indication of phosphatidic acid-specific phospholipase C activity (DAG/PA) in the crude spent media. In conclusion, here we determine there to be the presence of phosphatidylglycerol, phosphatidylinositol and phosphatidylserine specific phospholipase A1/A2 activity in the crude spent media from *S*.*aureus* JE2 and MN8 (lyso/ PL red border; Fig 9). We also determined that the increase in diacylglycerol previously observed may be due to the degradation of phosphatidic acid resulting from PLC activity (DAG/PA blue border; Fig 9). We were also able to detect the presence of sphingomyelinase activity (Cer/SM) in the strain *S*. *aureus* COL that would result in the degradation of sphingomyelin and increase in ceramides, which is previously unreported. There is also evidence of medium to high levels of PE specific phospholipase D activity (PA/PE) that would account for the increase in PA in *S*. *aureus* JE2 and MN8 treated groups. We were unable to explain the decrease in the PA to PI to ratio as there is no known enzyme to synthesize PI from PA directly. We think this ratio to be affected by the gross increase in PI (Fig 4) in the *S*.*aureus* JE2 and MN8 treated groups.

## Discussion

To test our hypothesis that virulent bacteria modulate host lipids to a greater extent than non-pathogenic and commensal bacteria, we compared the lipid modifying capabilities of *S*. *aureus* strains JE2, MN8, COL, and Newman with *S*. *epidermidis* RP62A and *S*. *carnosus* TM300. JE2 is a methicillin resistant strain of the USA300 pulsed field gel electrophoresis type. USA300 is the most common type isolated from community acquired MRSA infections and is also a common hospital-associated type [71]. USA300 strains are all *agr* group I and are considered highly virulent. MN8 is a methicillin sensitive USA200 isolate from a case of toxic shock, is a strong producer of the TSST-1 toxin, and is also considered highly virulent[55]. COL is a methicillin resistant strain and a clinical isolate but is *agr*-negative and does not produce alpha, beta, or delta hemolysin. Newman is a methicillin sensitive strain and was isolated from an infection secondary to a *Mycobacterium tuberculosis* osteomyelitis, and displays constitutive sensor histidine kinase (SaeS) activity[72,58]. *S*. *epidermidis* strain RP62A is a clinical isolate of a normally commensal staphylococcal species and *S*. *carnosus* TM300 is a non-pathogenic isolate that has been rigorously tested for safe use as a sausage starter culture.

Traditional lipase assays indicated substantively greater lipase activity in spent media from *S*. *aureus* strains JE2 and MN8 relative to other strains. This is suggestive of a correlation between virulence and lipase activity, however, virulence of *S*. *aureus* strains varies significantly based on the animal model in which it is tested. Similarly, the role of lipase activity and the roles of other virulence factors, may differ based on the tissue infected in the human host. The plate based assay (Fig 2A) which uses egg yolk as a substrate is abundant in phospholipids like phosphatidylcholine, clearly demonstrated the ability of *S*. *aureus* (JE2 and MN8) to hydrolyze phospholipids and was confirmed in our mass spectrometry based approach (Fig 3). Lipid hydrolysis by lipases falls into two categories; hydrolysis of water soluble substrates (esterase) and water insoluble substrates (lipase) [73]. The egg yolk agar plate is a sensitive method for the identification of lipase activity (insoluble substrates) and the DTNB colorimetric assay[53] is sensitive for the detection of lipase activity against water soluble substrates (or esterase activity)[73]. The shotgun lipidomic assay developed in this study was sensitive enough to detect both kinds of activities, and identified lipid substrates that may be biologically relevant in *S*. *aureus* pathogenesis.

Characterization of the host lipidome and greater appreciation of lipids in pathological processes has been made possible by mass spectrometry based lipidomic approaches. In our MS/MS^ALL^ based shotgun lipidomic approach we corroborate the hydrolysis of phosphatidylcholine seen on the plate-based assay (Figs 2A and 5B). Based on the colorimetric assay, we conclude that spent media from COL, Newman, *S*. *epidermidis*, and *S*. *carnosus* cultures not only lack lipase activity but also lack esterase activity (Fig 2B). The results were expected as *S*. *carnosus*, which is used as a sausage starter culture [51] has a predominantly fermentative metabolism based on the utilization of short chain fatty acids. Investigating the regulation of secreted lipid modifying enzymes was beyond the scope of this study. However, the Agr system plays a known regulatory role in the secretion of many virulence factors including the lipase Geh [74], and COL, a strain deficient in *agr*, failed to exhibit many of the lipid modifications exhibited by JE2 and MN8. Thus, Agr could potentially play a global role in the secretion of lipases. Deficiency of lipase activity in strain Newman may be attributed, in part, to the inactivation of Geh by phage integration[4].

In our initial hypothesis, we expected the lipid modifying capabilities of *S*. *aureus* JE2 and MN8 to be limited to the hydrolysis of lipids, i.e. lipase activity. However, based on recent reports [11,17] and our results (Figs 6 and 7) we conclude that extracellular enzymes from *S*. *aureus* have the ability to esterify free fatty acids to PLs in addition to lipid hydrolysis that is not seen in *S*. *aureus* Newman and *S*. *carnosus*. The ability of *S*. *aureus* to esterify host derived free fatty acids to phospholipids has been shown to regulate virulence factor production in the bacteria [37,38]. The downstream effects of this regulation may play a role in the fitness of the pathogen in-vivo [10,38,39]. Though previous studies have shown host derived fatty acids to be incorporated into host lipids[37], this is the first to our knowledge that demonstrates the ability of *S*. *aureus* to produce the fatty acids from mammalian lipids and further re-esterify them back to acceptor lipids abundant such as phosphatidylglycerol and phosphatidylinositol in the bacterial cell membrane (Figs 6 and 7). *S*. *aureus* demonstrates specificity for the classes of phosphatidylethanolamine, phosphatidylglycerol and phosphatidylserine (Figs 5A and 7) for re-esterification, suggesting that *S*. *aureus* uses major membrane lipid classes as a source for the production of host derived free fatty acids and cholesteryl esters, phosphatidylinositol, and lysophosphatidylinositol (Fig 5) as recipients for these fatty acids. Though it is possible that the increase in diacylglycerol is due to the esterification of free fatty acids to monoacylglycerol (MAG) known to be growth inhibitory to *S*. *aureus* [75] by fatty acid kinases, it is hard to draw this conclusion as we did not look at monoacylglycerol in our analysis and a separate experiment including only monoacylglycerol, diacylglycerol and triacylglycerol is required to determine this flux.

The medium chain fatty acids released following treatment with *S*. *aureus* spent media (Fig 8; S3 Fig) can be detrimental to the growth of the bacteria in pure culture[12]. However, this ability of *S*. *aureus* to esterify fatty acids to PLs suggests a mechanism by which the bacteria are able to detoxify such an environment. In our experiments we find that medium chain fatty acids reported in previous studies were esterified to lipids as denoted by the increase in their relative abundance after treatment with spent media from *S*. *aureus* strains JE2 and MN8 (Figs 6 and 7). We find these increases to be restricted to lipids containing oleic acid (18:1) in the classes of cholesteryl ester, phosphatidylcholine, and phosphatidylinositol. No increases were seen in lipids associated with lipids containing fatty acids with more than 2 degrees of unsaturation and we believe the FakA mediated esterification of fatty acids[11] to phospholipids to be restricted by the degree of unsaturation and not the length of the free fatty acid. The absence of this modification in the heat-inactivated controls of *S*. *aureus* suggests the origin of the molecule responsible for these modifications to be proteinaceous in nature and not due to the heat inactivation process. Though the exact enzymes responsible for the observed modifications cannot be determined, we were able to identify the interaction of staphylococcal lipid modifying enzymes with previously unreported lipid classes like cholesteryl ester, phosphatidylcholine, phosphatidylethanolamine, phosphatidylinositol and phosphatidylserine. In addition to the interaction of phospholipids by *S*. *aureus* JE2 and MN8 we detected increases in the class of ceramides in groups treated with spent media from *S*. *epidermidis* and *S*. *aureus* COL (Fig 5B). While there has been no prior indication of the presence of lipase activities contributing to ceramide synthesis in *S*. *aureus* or *S*. *epidermidis*, we believe these changes to be related to the function of yet undiscovered enzyme involved in the de novo biosynthesis of ceramides. The possibility of ceramide synthesis from sphingomyelin may be argued against based on the absence of degradation of sphingomyelin in *S*. *aureus* COL- and *S*. *epidermidis-* treated groups (Figs 5B and 6) and less than two fold increased in the Cer/SM ratio indicative of sphingolmyelinase activity (Fig 9). This is also well demonstrated by a meager increase in Cer/SM ratio, which suggests that ceramide synthesis is independent of the abundance of sphingomyelin. The increase in the relative abundance of ceramides though unexpected may indicate the presence of a mutualistic relationship between a commensal microbe (*S*. *epidermidis*) that colonizes the skin and helps maintain the cellular integrity by maintaining ceramide levels. This is also in contrast to pathogenic *S*. *aureus* that express beta-hemolysin, a sphingomyelinase C[25], to hydrolyze sphingomyelin to compromise endothelial integrity[76]. The absence of sphingomyelin degradation in our experiment suggests that *S*. *aureus* strains JE2 and MN8 have reduced beta-hemolysin production, at least under the conditions used in this study (Fig 5B).

With our mass spectrometry-based approach we were able to not only identify overall modifications in lipid profiles but also provide the identification of molecular lipid species and their fatty acid composition that may be important for its pathogenesis. This sheds light on the existence of yet unknown lipase activities (Fig 9) that merit further identification. A limitation of our approach was the inability, based on current experimental setup, to perform absolute quantification of lipids and future work could be directed towards absolute quantification of lipids and determining the origin and target of host derived fatty acids and their partner bacterial enzymes by flux based lipidomics. In our approach while we assert the presence of novel lipase activities in crude spent media (Fig 9) we were unable to identify the enzyme responsible for the observed activity. Further work to purify and characterize these enzymes that contribute to phospholipase D and other phospholipase A1/A2 is required. Another potential limitation is that staphylococci may alter their secretome in response to changes in their environment. Thus, isolates that appeared to have little lipase activity in this study, could express lipases under different conditions. Strengths of the study include the use of both modes of ionization to identify and verify the sum lipid composition. We also demonstrate a simple approach to assay for extracellular lipase activity in the absence of prior information about putative lipases. In conclusion we believe such comprehensive lipidomic profiling to provide a top down approach for identifying specific components of lipid metabolism, utilized by pathogenic bacteria, which may be further targeted for the management of increasingly drug resistant infections and identification of novel lipid modifying virulence factors conserved among pathogenic bacteria.

## Supporting information

S1 Fig Effect of *S*. *aureus* supernatant on neutral and sphingolipids. High resolution images with naming of lipids in the Unidirectional Hierarchical clustering of significant neutral lipid (Fig 4A) species. PBS; PBS control, *JE2; S*. *aureus JE2*, *MN8*: *S*.*aureus MN8*, *COL; S*. *aureus (agr-)*, *NEW; S*. *aureus Newman (constitutive saeS)*, *EPI; S*. *epidermidis RP62A*, *CAR; S*. *carnosus TM300*.
(TIF)

S2 Fig Effect of *S*. *aureus* supernatant on phospholipids. High resolution images with naming of lipids in the Unidirectional Hierarchical clustering of significant phospholipid species (Fig 4B). PBS; PBS control, *JE2; S*. *aureus JE2*, *MN8*: *S*.*aureus MN8*, *COL; S*. *aureus (agr-)*, *NEW; S*. *aureus Newman (constitutive saeS)*, *EPI; S*. *epidermidis RP62A*, *CAR; S*. *carnosus TM300*.
(TIF)

S3 Fig Comparison of TOFMS chromatograms for each treatment. TOFMS chromatograms for the heart extracts were analyzed with reference to the PBS control after treatment with each bacterial supernatant. Black trace- PBS, Red trace- treatment. *m/z* range 0–1000, Y- axis is presented as %Intensity to the largest peak in the graph. +TOFMS- positive mode accurate mass spectra from MS1, -TOFMS- negative mode accurate mass spectra from MS1. PBS; PBS control, *JE2; S*. *aureus JE2*, *MN8*: *S*.*aureus MN8*, *COL; S*. *aureus (agr-)*, *NEW; S*. *aureus Newman (constitutive saeS)*, *EPI; S*. *epidermidis RP62A*, *CAR; S*. *carnosus TM300*.
(TIF)

S1 File Supplement significant lipid plots. All plots generated for significant lipid modulations by the strains can be found in this file. This file also includes the results for analysis of effect of treatment on fatty acid composition of lipids and complete list of lipid species modulated by the strains in this study.
(HTML)

S2 File Significant lipids Kruskal-Wallis. Statistical output of all lipids determined to be significant by Kruskal-Wallis (column; p-value) and Benjamini Hochberg correction (column; p.adj (BH)).
(CSV)
