# Supplementary figures and images for: Untargeted lipidomic analysis to broadly characterize the effects of pathogenic and non-pathogenic staphylococci on mammalian lipids
Source: PLoS One. 2018 Oct 31;13(10):e0206606. doi: 10.1371/journal.pone.0206606 (PMC6209338; doi:10.1371/journal.pone.0206606)

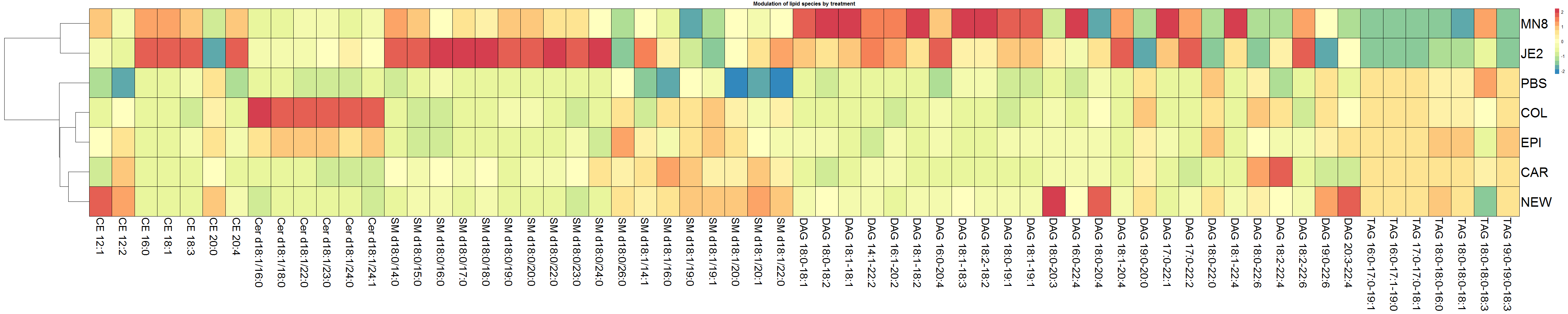

Supplement: S1 Fig — High resolution images with naming of lipids in the Unidirectional Hierarchical clustering of significant neutral lipid (Fig 4A) species. PBS; PBS control, JE2; S. aureus JE2, MN8: S.aureus MN8, COL; S. aureus (agr-), NEW; S. aureus Newman (constitutive saeS), EPI; S. epidermidis RP62A, CAR; S. carnosus TM300. (TIF) [file pone.0206606.s001.tif]

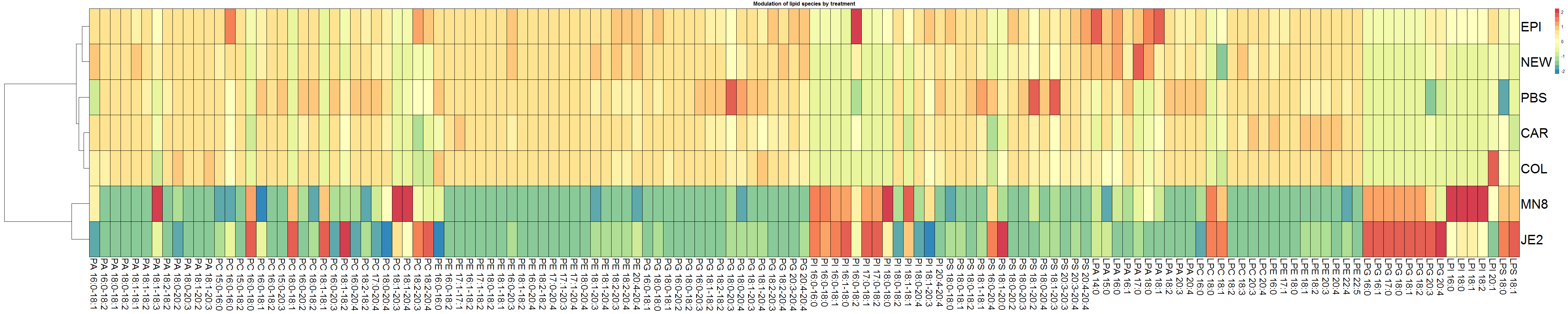

Supplement: S2 Fig — High resolution images with naming of lipids in the Unidirectional Hierarchical clustering of significant phospholipid species (Fig 4B). PBS; PBS control, JE2; S. aureus JE2, MN8: S.aureus MN8, COL; S. aureus (agr-), NEW; S. aureus Newman (constitutive saeS), EPI; S. epidermidis RP62A, CAR; S. carnosus TM300. (TIF) [file pone.0206606.s002.tif]

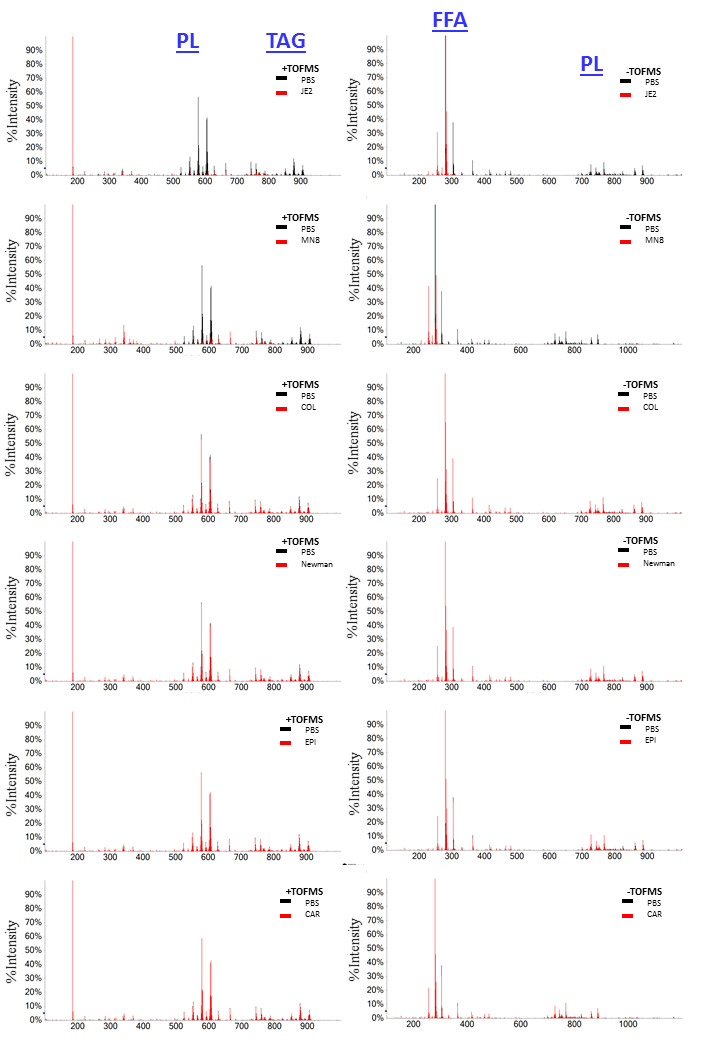

Supplement: S3 Fig — TOFMS chromatograms for the heart extracts were analyzed with reference to the PBS control after treatment with each bacterial supernatant. Black trace- PBS, Red trace- treatment. m/z range 0–1000, Y- axis is presented as %Intensity to the largest peak in the graph. +TOFMS- positive mode accurate mass spectra from MS1, -TOFMS- negative mode accurate mass spectra from MS1. PBS; PBS control, JE2; S. aureus JE2, MN8: S.aureus MN8, COL; S. aureus (agr-), NEW; S. aureus Newman (constitutive saeS), EPI; S. epidermidis RP62A, CAR; S. carnosus TM300. (TIF) [file pone.0206606.s003.tif]
