# Supplementary material for: Untargeted lipidomic analysis to broadly characterize the effects of pathogenic and non-pathogenic staphylococci on mammalian lipids
Source: PLoS One. 2018 Oct 31;13(10):e0206606. doi: 10.1371/journal.pone.0206606 (PMC6209338; doi:10.1371/journal.pone.0206606)
Supplement: S1 File — All plots generated for significant lipid modulations by the strains can be found in this file. This file also includes the results for analysis of effect of treatment on fatty acid composition of lipids and complete list of lipid species modulated by the strains in this study. (HTML) [file pone.0206606.s004.html]

Supplementary figures and plots-GKN


# Supplementary figures and plots-GKN

# Distribution of lipid species after filtering the dataset (>30cps and <75% zeros)

```
##  [1] "PBS" "PBS" "PBS" "PBS" "PBS" "PBS" "SA"  "SA"  "SA"  "SA"  "SA" 
## [12] "SA"  "MN"  "MN"  "MN"  "MN"  "MN"  "MN"  "COL" "COL" "COL" "COL"
## [23] "COL" "COL" "NEW" "NEW" "NEW" "NEW" "NEW" "EPI" "EPI" "EPI" "EPI"
## [34] "EPI" "EPI" "CAR" "CAR" "CAR" "CAR" "CAR" "CAR" "SAB" "SAB" "SAB"
## [45] "SAB" "SAB" "SAB" "MB"  "MB"  "MB"  "MB"  "MB"  "MB"  "CB"  "CB" 
## [56] "CB"  "CB"  "CB"  "CB"  "NB"  "NB"  "NB"  "NB"  "NB"  "NB"  "EB" 
## [67] "EB"  "EB"  "EB"  "EB"  "EB"  "SCB" "SCB" "SCB" "SCB" "SCB" "SCB"
```

```
## Warning: package 'bindrcpp' was built under R version 3.3.3
```

# Cholesteryl esters (CE)

# Ceramides (Cer)

# Sphingomyelin (SM)

# Diacylglycerol (DAG)

# Triacylglycerol (TAG)

# Phosphatidylcholine (PC)

# Phosphatidylethanolamine (PE)

# Phosphatidylglycerol (PG)

# Phosphatidylinositol (PI)

# Phosphatidylserine (PS)

# Phosphatidic acid (PA)

# lysophosphatidylcholine (LPC)

# lysophosphatidylglycerol (LPG)

# lysophosphatidic acid (LPA)

# lysophosphatidylethanolamine (LPE)

# lysophosphatidylinositol (LPI)

# lysophosphatidylserine (LPS)

# class distribution of summed fragmet mol% data

## Plot class based distribution to total lipid for groups

# Determine fatty acid distribution from raw files after filtering for peak intensity

# Plot FA based distribution to total lipid for groups

```
## Using Group as id variables
```

```
## Scale for 'y' is already present. Adding another scale for 'y', which
## will replace the existing scale.
```

# Free Fatty Acid/ TAG ratio
